# Simultaneously Regulating Electrochemical Corrosion Behavior and Wettability of Magnesium–Neodymium Alloy by Self-Layered Chemical Conversion Coating

**DOI:** 10.3390/ma17122815

**Published:** 2024-06-09

**Authors:** Keke Yang, Yulian Kuang, Bingqian Xu, Changyang Liu, Guosong Wu

**Affiliations:** College of Mechanics and Materials, Hohai University, Nanjing 211100, China; 211308020025@hhu.edu.cn (K.Y.); 202309021104t@cqu.edu.cn (Y.K.); bingqianqian@outlook.com (B.X.); liuchangyang@hhu.edu.cn (C.L.)

**Keywords:** magnesium, coating, surface, corrosion, wettability

## Abstract

Rapid corrosion in aqueous solutions of magnesium alloys is one of the major obstacles to their wide application, and coating plays a crucial role in their corrosion protection. Recently, protection- and function-integrated coatings have attracted much attention in the research field of magnesium alloys. In this work, a simple chemical conversion process is proposed to fabricate a composite coating on a magnesium–neodymium alloy through immersion in an aqueous solution made of Ca(OH)_2_ and NaHCO_3_. After the immersion process, a coating consisting of two spontaneously formed layers is acquired. The top flower-like layer is composed of Mg_5_(OH)_2_(CO_3_)_4_∙4H_2_O, Mg(OH)_2_ and CaCO_3_, and the inner dense layer is speculated to be Mg(OH)_2_. Electrochemical impedance spectroscopy, polarization tests, and hydrogen evolution are combined to evaluate the corrosion resistance in simulated body fluid, simulated seawater solution, and simulated concrete pore solution, which reveals that the coated sample has better corrosion resistance than the uncoated one. After the coated sample is modified with fluorinated silane, a water-repellent surface can be achieved with an average water contact angle of 151.74° and a sliding angle of about 4°. Therefore, our results indicate that effective corrosion protection and potential self-cleaning ability have been integrated on the surface of the magnesium alloy in this study. In addition, the formation mechanism of the self-layered coating is discussed from the viewpoint of the interaction between the substrate and its external solution.

## 1. Introduction

The energy crisis seriously affects the development of modern society and reducing energy consumption is one of the effective ways to deal with the problem. Today, the concept of lightweight design, which aims to reduce energy demand, improve energy efficiency, and reduce carbon dioxide emissions, has been accepted in modern industries [1,2]. Magnesium is one of the lightweight metals with the most potential since the density of magnesium is only a quarter of that of iron and two-thirds of that of aluminum [3]. Moreover, magnesium and its alloys have high specific strength and stiffness, good damping ability, excellent machinability and dimensional stability, superior electromagnetic shielding performance, good biocompatibility, etc. [4,5,6], attracting considerable attention in aerospace, automotive, biomedical and other fields [7,8,9]. However, their poor corrosion resistance in aqueous solutions still hinders their wider application since magnesium has a negative potential of −2.37 V [10] and the natural oxide film or hydroxide film formed on magnesium has no protective effect [4]. Even though magnesium alloys can be used as biodegradable metals due to their intriguing biodegradation and biosorption, their rapid biodegradation rate in a physiological environment is not acceptable [11].

Usually, the corrosion resistance of magnesium alloys can be improved by three ways, i.e., alloying [12], microstructure regulation [13,14], and surface modification [15,16]. Among these strategies, it is relatively economical to introduce corrosion-resistant coatings on magnesium alloys as a physical barrier, isolating the substrates from the aggressive environments. Chemical conversion coatings including chromate coatings [17,18], phosphate coatings [11,19], carbonate coatings [20,21], stannate coatings [22,23], permanganate coatings [24] and phytic acid coatings [25] have been commonly used as protective barriers. Among them, chromate coating is commonly used as an anti-corrosion coating for magnesium alloy because of its excellent corrosion resistance, self-healing performance, and relatively mature process. However, Cr^6+^ is highly toxic and poses a significant threat to humans and the environment [17,26]. Therefore, its industrial applications have been restricted. At present, it is still urgent to find a more non-toxic and environmentally friendly coating for corrosion protection of magnesium alloys.

Zhang et al. [27] proposed a concept involving a pre-corrosion treatment to produce a double-barrier structure on AZ80 Mg alloys to resist aqueous corrosion. They first used a one-step hydrothermal process in water to obtain a hierarchical coating composed of an inner compact hydroxide layer and top uniform Mg-Al LDH micro-sheets. After subsequent modification with fluorinated silanes, a Cassie-state superhydrophobic surface can be achieved with enhanced long-term corrosion resistance and potential self-cleaning ability. Obviously, it is an effective way to prepare a corrosion-protection- and surface-functionalization-integrated coating on the AZ80 magnesium alloy. Besides the self-cleaning ability, superhydrophobic surfaces have other functions, such as anti-contamination and anti/de-icing [28]. Therefore, there is great potential in developing this coating process based on pre-corrosion.

Deepa B. Prabhu et al. [29] prepared MgCO_3_∙3H_2_O coatings by immersing a Mg-4Zn alloy in a saturated NaHCO_3_ solution at room temperature, which exhibited a biomineralization effect. Yao et al. [30] immersed a magnesium–neodymium alloy in potassium dihydrogen phosphate solution at 50 °C for 4 h and obtained a layer of uniform MgHPO_4_·3H_2_O coating with a thickness of about 20 µm, which improves the corrosion resistance of the Mg alloy in a Cl^−^-containing simulated concrete pore solution. Wang et al. [31] immersed an AZ80 alloy in a simulated concrete pore solution made of saturated Ca(OH)_2_ solution for 30 days and found that it has good corrosion resistance due to a spontaneously formed protective Mg(OH)_2_ film. Therefore, this indicates that the pre-corrosion strategy is feasible to improve the corrosion resistance of magnesium alloys. However, the application of this concept is still in the initial stage, and it is necessary to continue to expand the scope of exploration and deeply understand the relevant mechanisms behind the immersion process, which can lead us to finally form a complete knowledge system for designing corrosion-protection- and surface-functionalization-integrated coatings in the future. In this study, we attempt to treat a magnesium–neodymium alloy with a mixed solution created by a saturated Ca(OH)_2_ solution and 0.1 mol/L NaHCO_3_ solution. In addition, the corrosion resistance of treated samples is evaluated in simulated physiological solutions, simulated seawater solutions, and simulated concrete pore solutions. Moreover, the associated mechanisms in this study are discussed.

## 2. Materials and Methods

As-cast Mg-3.3 Nd (wt.%) alloys were used as substrate materials in this study and cut into cubic samples with sizes of 10 mm × 10 mm × 5 mm. They were first mechanically ground with SiC sandpaper up to #2000, and then ultrasonically cleaned in ethanol for 3 min at room temperature. Equipment that can provide a constant temperature water bath was used for coating preparation. Then, 20 mL of saturated Ca(OH)_2_ solution and 40 mL of 0.1 mol/L NaHCO_3_ solution were prepared, and both were preheated at 80 °C for 5 min. The samples were first placed in the saturated Ca(OH)_2_ solution, and then 40 mL of 0.1 mol/L NaHCO_3_ solution was slowly poured. Next, the beaker containing the mixed solution and samples was heated at 80 °C for 6 h. After this process, the samples were removed from the solution and rinsed with ethanol and dried in air. After coating, surface modification with fluorinated silane was conducted as follows. First, a mixed solution was prepared by perfluorododecyltriethoxysilane (C_16_F_17_H_19_O_3_Si, PFDS), deionized water, and ethanol with a mass ratio of 1:4:45. Then, the uncoated and coated samples were soaked in the solution for 2 h, respectively. After being heated in an oven at 140 °C for 1 h, they were dried in air. In this paper, the as-received sample was denoted as uncoated and the coated sample was named as coated. After surface modification with PFDS, these two samples were denoted as PFDS-uncoated and PFDS-coated, respectively.

The coating morphology and chemical composition of the coating sample were observed by scanning electron microscopy (SEM, Hitachi Regulus 8100, Tokyo, Japan) equipped with X-ray energy-dispersive spectroscopy (EDS). Here, the samples were coated by sputtered gold before SEM observation. The phase composition of the substrate and coated samples were studied by X-ray diffraction (XRD, Rigaku SmartLab SE, Tokyo, Japan) with Cu Ka radiation at a scan rate of 2°/min. The chemical composition and chemical state of the coating were analyzed by X-ray photoelectron spectroscopy (XPS, Thermo Scientific K-Alpha Waltham, MA, USA) with an Al Kα X-ray radiation source. In addition, transmission electron microscopy (TEM, FEI Talos F200X G2, Waltham, MA, USA) combined with X-ray energy-dispersive spectroscopy (EDS) was applied to investigate the phase composition and chemical composition of the powder scratched from the coating. The powder was first sonicated in ethanol for 5 min to avoid agglomeration and then used for TEM observation.

In this study, three kinds of solutions were used to evaluate the corrosion resistance of the coated samples. A 0.9 wt.% NaCl solution heated at 37 °C was utilized to simulate the physiological environment. A simulated seawater solution used in this study was composed of 24.53 g/L NaCl, 4.09 g/L Na_2_SO_4_, 11.11 g/L MgCl_2_, 1.16 g/L CaCl_2_, 0.04 g/L SrCl_2_, 0.70 g/L KCl, 0.20 g/L NaHCO_3_ and 0.1 g/L KBr [32]. In addition, a saturated Ca(OH)_2_ solution was proposed to simulate a concrete pore solution and, in order to study the effect of Cl^−^, sodium chloride (NaCl) was added into the saturated Ca(OH)_2_ solution to increase the concentration of NaCl up to 2.5 wt.%. Electrochemical measurements involving potentiodynamic polarization and electrochemical impedance spectrum (EIS) measurements were conducted using an electrochemical workstation (Gamry, Reference 600+). A saturated calomel electrode (SCE) was used as the reference electrode, and the counter electrode was made of platinum sheets. The test sample with an exposed area of 10 mm × 10 mm acted as the working electrode. After being soaked in a 100 mL solution for 1 h, the potential was scanned from the cathodic region to the anodic region at a rate of 1 mV/s to obtain the polarization curves. The corrosion potential and corrosion current density were obtained using the cathode Tafel extrapolation method. The data of EIS measurement were recorded from 100 kHz to 0.1 Hz with a 5 mV sinusoidal perturbing signal at the open-circuit potential. In addition, the corrosion resistance was also estimated from hydrogen evolution measurements in the aforementioned solutions. The samples were sealed with acrylic-based resin with an exposed area of 10 mm × 10 mm and the method of hydrogen collection was carried out as referenced in the literature [33]. The static water contact angle (CA) and sliding angle (SA) of the samples were measured with a 4 μL ultrapure water droplet on a contact angle measuring instrument (POWEREACH, JC2000D1,Shanghai, China).

## 3. Results and Discussion

**Figure 1:**
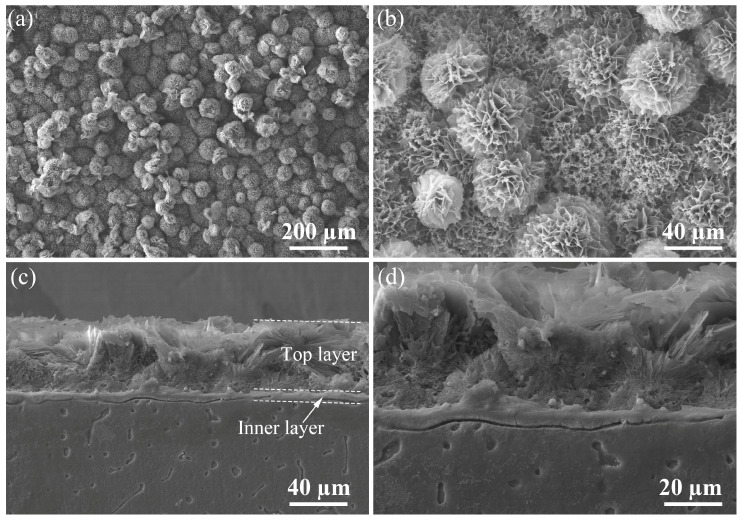
SEM images showing the surface and cross-section morphologies of the coated sample: (**a**) the surface observed under lower magnification, (**b**) the surface observed under higher magnification, (**c**) the cross-section observed under lower magnification, (**d**) the cross-section observed under higher magnification.

Figure 1 shows SEM micrographs of the surface and cross-section morphologies of the coated sample. As shown in Figure 1a, the surface of the coated sample is uniformly covered by plush micro-spheres. Figure 1b shows the magnified view of the spheres, which reveals that the flower-like sphere consists of many micro-sheets. In addition, it is found that many cubic particles are scattered between micro-sheets. Figure 1c,d shows the cross-sectional morphology of the coating with different magnifications. Here, the thickness of the coating can be calculated to be 53.62 ± 3.87 µm. Interestingly, the coating is composed of two layers: the top layer is related to the sphere-like array and the inner layer is relatively compact. It can be further observed that the inner layer is much thinner than the top layer.

**Figure 2:**
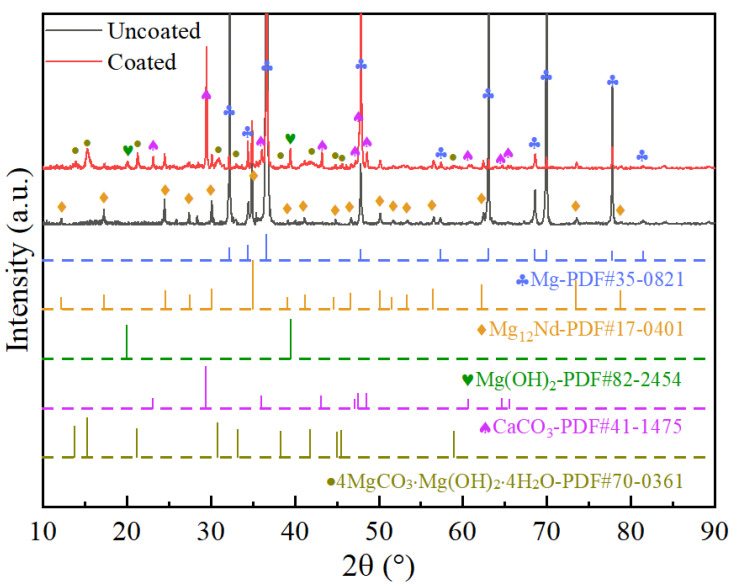
XRD patterns of the coated and uncoated samples.

**Figure 3:**
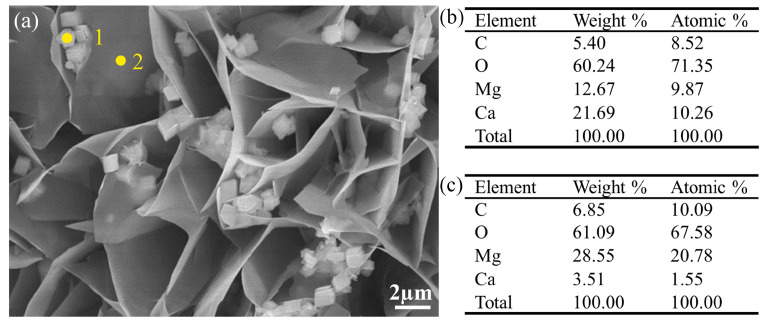
(**a**) SEM image of the surface morphology of the coated sample; (**b**) EDS result of point 1; (**c**) EDS result of point 2.

**Figure 4:**
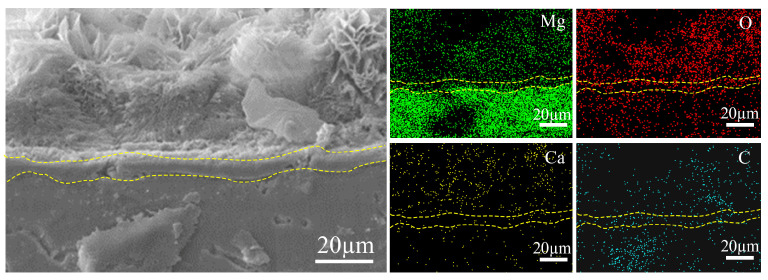
SEM image of the cross-section morphology of the coated sample and the corresponding EDS mappings.

Figure 2 shows the XRD results of the coated and uncoated samples. By comparison, it is determined that the obtained coating is mainly composed of Mg(OH)_2_, CaCO_3_, and Mg_5_(OH)_2_(CO_3_)_4_∙4H_2_O and the peaks of Mg and Mg_12_Nd originate from the substrate. EDS results were further utilized to analyze the chemical composition of the coating in this study. The results shown in Figure 3 and Figure 4 reveal that the coating mainly consists of Mg, Ca, C, and O. It can be found in Figure 3 that the concentration of Ca in point 1 is much higher than that in point 2. As shown in Figure 4, it is corroborated by EDS mapping that the coating consists of two layers. By combining the elemental distribution with the XRD results, it is speculated that the bottom layer marked with a dash line is possible to be Mg(OH)_2_.

**Figure 5:**
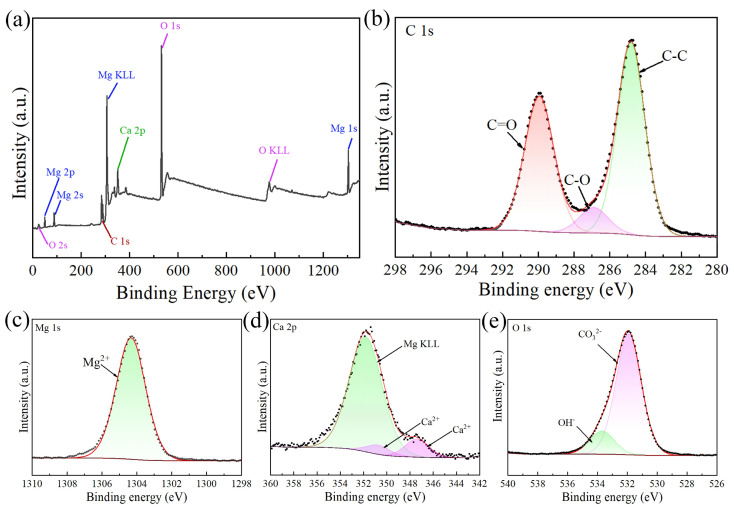
XPS spectra of the coated sample: (**a**) survey spectrum; (**b**) high-resolution spectrum of C1s; (**c**) high-resolution spectrum of Mg 1s; (**d**) high-resolution spectrum of Ca 2p; (**e**) high-resolution spectrum of O 1s.

Figure 5 presents the results of the XPS test performed on the coated sample, which can further determine the chemical binding state of coating elements. As shown in Figure 5a, the XPS survey spectrum reveals the existence of elements Mg, Ca, C, and O on the surface of the coating. This is also consistent with the aforementioned EDS result. High-resolution XPS spectra are deconvoluted and shown in Figure 5b–e. The three components with binding energies of 284.8 eV, 286.56 eV, and 290.01 eV are obtained by deconvolution of C 1s in Figure 5b, which belong to C-C, C-O, and C=O [34,35], respectively. Figure 5c shows that the peak of the high-resolution spectrum of Mg 1s appears at 1304.51 eV, which can be attributed to Mg^2+^ rather than metallic magnesium [36]. The deconvolution of Ca 2p shown in Figure 5d indicates that the actual peak stems from Ca^2+^ and the Auger peak of Mg. Similarly, the deconvolution of O 1s shown in Figure 5e reveals the presence of CO_3_^2−^ and OH^−^.

**Figure 6:**
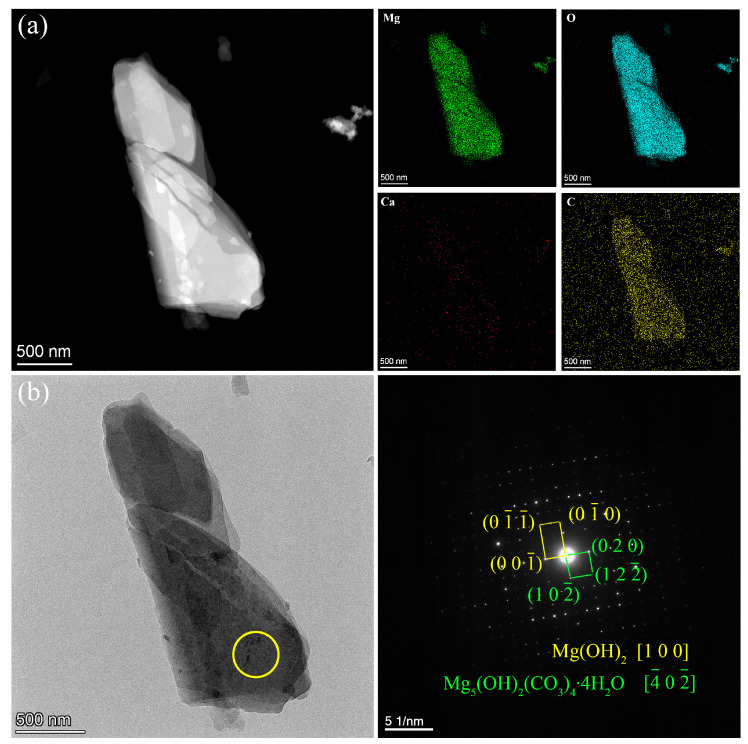
TEM images of the flaky powder from the coating: (**a**) HAADF image and corresponding EDS mappings. (**b**) SAED pattern measured at the circle area in TEM image.

**Figure 7:**
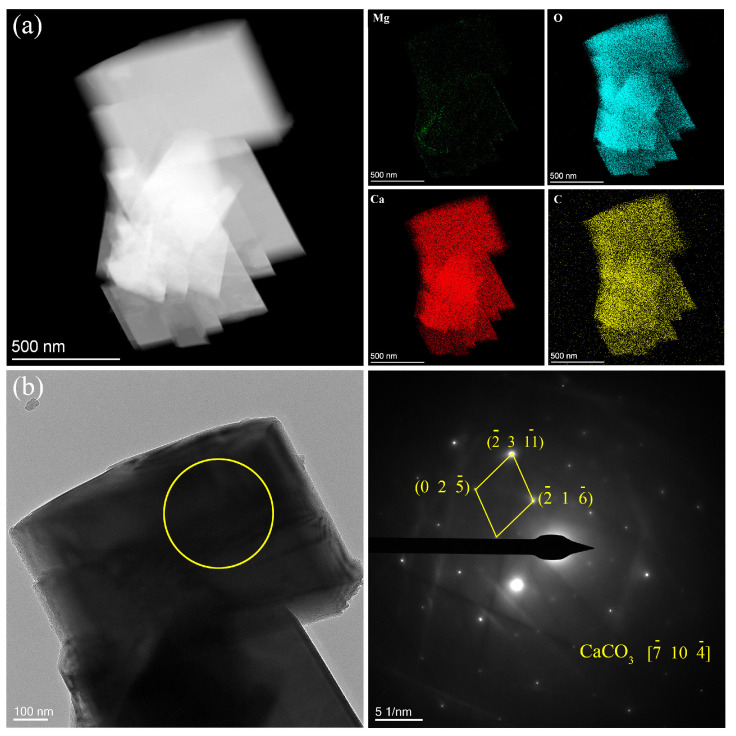
TEM images of the blocky powder from the coating: (**a**) HAADF image and corresponding EDS mappings. (**b**) SAED pattern measured at the circle area in TEM image.

In order to further analyze the detailed structural information and composition of the coating, the powder scratched from the coating was characterized by TEM with high-angle annular dark field (HAADF) mode and selected area electron diffraction (SAED) in this study. Two forms of powders are shown in Figure 6 and Figure 7. As shown in Figure 6a, EDS mappings of the flaky powder exhibit the elemental distribution of Mg, O, Ca, and C, which shows that the concentration of Ca is very low. Figure 6b shows the SAED diffraction pattern measured in the yellow circle area of the bright field TEM image, which matches the crystal data of Mg(OH)_2_ and Mg_5_(OH)_2_(CO_3_)_4_∙4H_2_O. Figure 7a shows the HAADF image and the corresponding EDS mappings of the blocky powder scratched from the coating. Here, the respective content of Ca, O, and C is much higher than that of Mg. Figure 7b shows the SAED pattern collected at the circle area in the bright field TEM image, which matches well with the crystal data of CaCO_3_.

Based on the aforementioned results, it can be concluded that a self-layered composite coating can be successfully achieved on the magnesium–neodymium alloy via a simple immersion process with a mixed solution of saturated Ca(OH)_2_ solution and 0.1 mol/L NaHCO_3_ solution. The top layer is mainly composed of CaCO_3_, Mg(OH)_2_ and Mg_5_(OH)_2_(CO_3_)_4_∙4H_2_O and the inner layer mainly consists of Mg(OH)_2_. The formation mechanism of the self-layered structure is speculated as follows: once the magnesium alloy contacts the solution, magnesium will react with the water according to the following formula: Mg + 2H_2_O → Mg(OH)_2_↓ + H_2_↑. Thus, the inner compact Mg(OH)_2_ layer is formed. Meanwhile, when the saturated Ca(OH)_2_ meets NaHCO_3_, several chemical reactions happen. First, HCO_3_^−^ ionized from NaHCO_3_ reacts with Ca(OH)_2_ based on the following formula: 2HCO_3_^−^ + Ca(OH)_2_ → CaCO_3_↓ + CO_3_^2−^ + 2H_2_O. Thus, CaCO_3_ precipitation is formed and CO_3_^2−^ is produced as well. Next, CO_3_^2−^ will react with free Mg^2+^ from the aqueous corrosion of magnesium substrate to produce Mg_5_(OH)_2_(CO_3_)_4_∙4H_2_O according to the following formula: 5Mg^2+^ + 4CO_3_^2−^ + 2OH^−^ + 4H_2_O → Mg_5_(OH)_2_(CO_3_)_4_∙4H_2_O↓. Therefore, the top layer is formed by the Mg_5_(OH)_2_(CO_3_)_4_∙4H_2_O micro-sheets embedded with microscale CaCO_3_.

**Figure 8:**
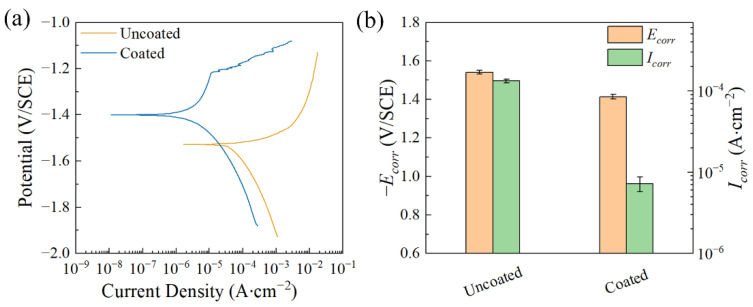
(**a**) Polarization curves of the samples after immersion in 0.9 wt.% NaCl solution at 37 °C for 1 h; (**b**) *E_corr_* and *I_corr_* calculated from the corresponding polarization curves.

**Figure 9:**
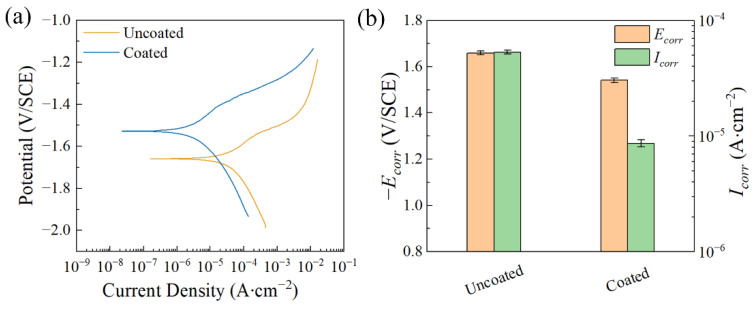
(**a**) Polarization curves of the samples after immersion in simulated seawater solution for 1 h; (**b**) *E_corr_* and *I_corr_* calculated from the corresponding polarization curves.

Figure 8a shows the polarization curves of the coated and uncoated samples in the 0.9 wt.% NaCl solution at 37 °C. The calculated *E_corr_* and *I_corr_* are shown in Figure 8b and Table 1. By comparison, the corrosion potential of the coated sample shifts positively and the corrosion current density is decreased by two orders of magnitude, ranging from (1.330 ± 0.072) × 10^−4^ A∙cm^−2^ to (7.548 ± 1.490) × 10^−6^ A∙cm^−2^. It is clearly indicated that the coating improves the corrosion resistance of the magnesium alloy in the 0.9 wt.% NaCl solution. Figure 9a presents the polarization curves of the coated and uncoated samples after being soaked in simulated seawater solution for 1 h. Figure 9b and Table 2 show the *E_corr_* and *I_corr_* corresponding to the polarization curves. It can be seen that the corrosion potential of the coated sample has shifted positively compared to that of the uncoated sample. The corrosion current density of the sample after coating decreases from (5.310 ± 0.019) × 10^−4^ A∙cm^−2^ to (8.650 ± 0.615) × 10^−6^ A∙cm^−2^. This implies that the coating can improve the corrosion resistance of the magnesium alloy in a simulated seawater solution.

**Figure 10:**
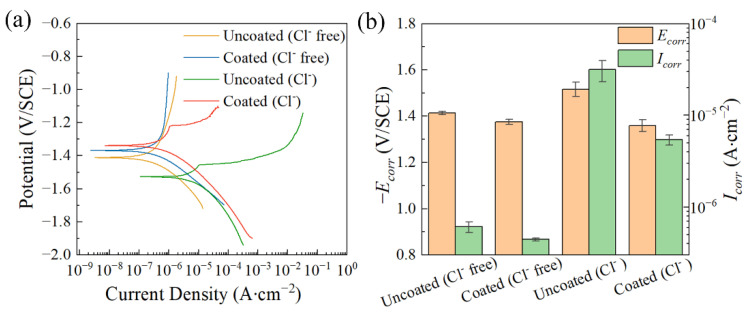
(**a**) Polarization curves of the samples after immersion in simulated concrete pore solution for 1 h; (**b**) *E_corr_* and *I_corr_* calculated from the corresponding polarization curves.

Figure 10 shows the polarization curves of the uncoated and coated samples after immersion in a simulated concrete pore solution for 1 h, as well as the calculated values of *E_corr_* and *I_corr_*. The details of the calculated values are shown in Table 3. The simulated concrete pore solution used in this test is a saturated Ca(OH)_2_ solution and the effect of Cl^−^ on corrosion behavior is investigated by adding NaCl. It can be seen that in the simulated concrete pore solution without Cl^−^, the corrosion potential of the coated sample is closed to that of the uncoated sample and the corrosion current densities of these two samples are of the same order of magnitude. However, in the simulated concrete pore solution containing Cl^−^, the corrosion potential of the coated sample shifts to the positive direction significantly and the corrosion current density decreases from (3.147 ± 0.816) × 10^−5^ A∙cm^−2^ in the uncoated sample to (5.414 ± 0.664) × 10^−6^ A∙cm^−2^ in the coated one. Therefore, this indicates that the coating can improve the corrosion resistance of the magnesium–neodymium alloy in a simulated concrete pore solution with Cl^−^.

**Figure 11:**
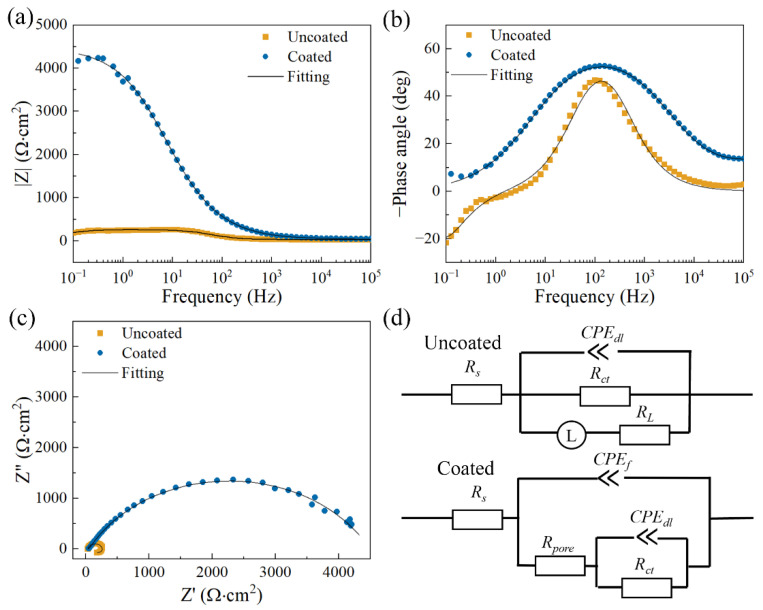
(**a**,**b**) Bode plots; (**c**) Nyquist plots; (**d**) the equivalent circuit plots of the samples after immersion in 0.9 wt.% NaCl solution for 1 h at 37 °C.

**Figure 12:**
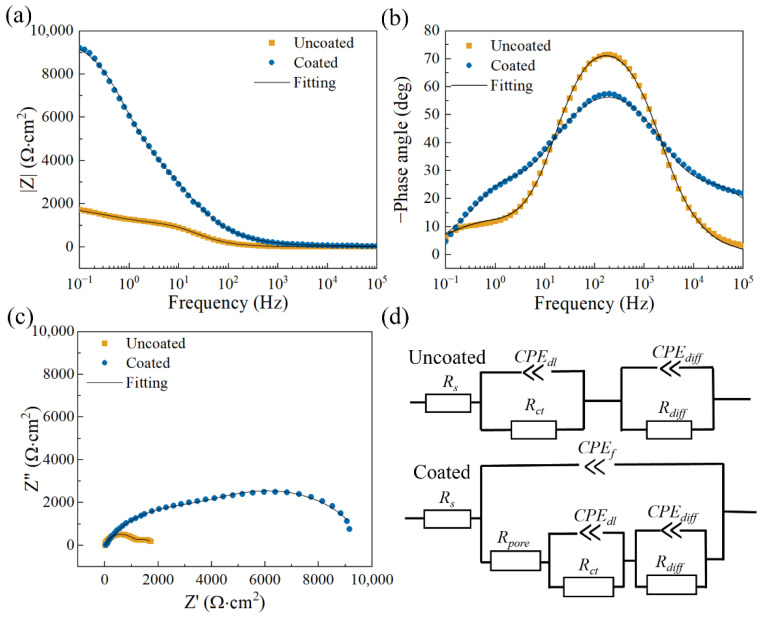
(**a**,**b**) Bode plots; (**c**) Nyquist plots; (**d**) the equivalent circuit plots of the samples after immersion in simulated seawater solution for 1 h.

**Figure 13:**
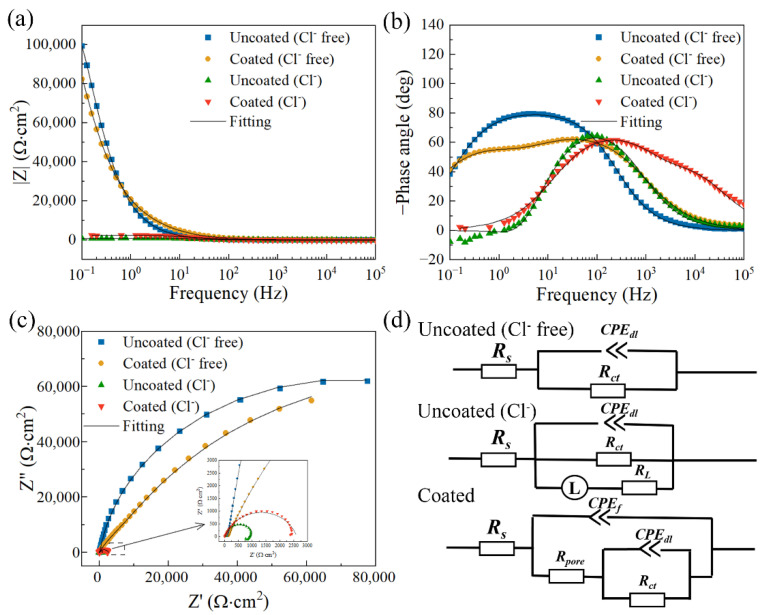
(**a**,**b**) Bode plots; (**c**) Nyquist plots; (**d**) the equivalent circuit plots of the samples after immersion in simulated concrete pore solution for 1 h.

The electrochemical impedance spectra of the uncoated and coated samples after being soaked in three kinds of solutions for 1 h are shown in Figure 11, Figure 12 and Figure 13. The experimental data and fitted data are represented by scatter plots and solid lines, respectively. Here, the fitting curves are calculated based on equivalent circuit models referring to studies [37,38].

Figure 11 shows the electrochemical impedance spectra of the uncoated and coated samples after being soaked in a 0.9 wt.% NaCl solution for 1 h at 37 °C. Figure 11a exhibits the Bode plots by impedance versus frequency and Figure 11b shows the Bode plots by phase angle versus frequency, Figure 11c gives the Nyquist plots. The equivalent circuit models for fitting are shown in Figure 11d. Roughly speaking, the larger the |Z| value at the lowest investigated frequency, the stronger the corrosion resistance [26,34]. It can be seen from Figure 11a that when the frequency is close to 0.1 Hz, the |Z| value of the coated sample is much larger than that of the uncoated sample. As shown in Figure 11c, both samples contain capacitive loop at a higher frequency, but the uncoated sample has an extra inductive loop in the low-frequency region. By comparison, the capacitive loop size of the coated sample is larger than that of the uncoated one. It is generally assumed that capacitance loops at higher frequencies can be attributed to thin film effects and charge transfer processes and the occurrence of induction loops at lower frequencies may stem from mass transfer processes [39,40]. Figure 11d shows two equivalent circuit models for the uncoated and coated samples. *R_s_*(*CPE_dl_R_ct_*(*LR_L_*)) is used to fit the EIS data of the uncoated sample and *R_s_*(*CPE_f_*(*R_pore_*(*CPE_dl_R_ct_*))) is applied to fit the EIS data of the coated sample. Here, R_s_ denotes the solution resistance, *CPE_f_* represents the capacitance of the thin film effect, *R_pore_* indicates the total resistance of pores in the thin film, *CPE_dl_* represents the electric double-layer capacitance, and *R_ct_* represents the charge transfer resistance. In addition, L and *R_L_* are used to describe the low-frequency inductance and related resistance, respectively. The fitted data are shown in Table 4. Usually, the polarization resistance R_p_ is reliable to evaluate corrosion resistance. Its reciprocal is directly proportional to the corrosion rate [41], i.e., the larger R_p_, the stronger the corrosion resistance of the material. According to the data in Table 4, $\frac{R_{ct}R_{L}}{R_{ct}+R_{L}}$ and $R_{pore}+R_{ct}$ are proposed to calculate the R_p_ value of the uncoated sample and the coated sample, respectively. The coated sample has a higher average R_p_ value of 4493.270 Ω·cm^2^ than the uncoated one, with an average R_p_ value of 91.824 Ω·cm^2^, indicating that it has better corrosion resistance.

Figure 12 shows the electrochemical impedance spectra of the uncoated and coated samples after being soaked in a simulated seawater solution for 1 h. The |Z|_f=0.1 Hz_ of the coated sample is much larger than the |Z|_f=0.1 Hz_ of the uncoated one, which implies that the coated sample has better corrosion resistance. As shown in Figure 12c, the Nyquist plots of the two samples consist of a high-frequency capacitive loop and an intermediate-frequency capacitive loop. As mentioned above, the capacitance loop at high frequencies can be attributed to the thin film effect and charge transfer process, while the intermediate-frequency capacitance loop may be related to diffusion. Obviously, the coated sample has larger capacitance loops, indicating stronger corrosion resistance. The two electric circuits in Figure 12d are used to fit the electrochemical impedance spectra of the uncoated and coated samples. *R_s_* is the solution resistance, *CPE_f_* represents the capacitance of the thin film effect, *R_pore_* represents the total resistance of pores in the thin film, *CPE_dl_* represents the electric double-layer capacitance, and *R_ct_* is the charge transfer resistance. In addition, *CPE_diff_* and *R_diff_* represent capacitance and resistance related to diffusion, respectively. Based on the fitted data in Table 5, $R_{ct}+R_{diff}$ and $R_{pore}+R_{ct}+R_{diff}$ are further used to calculate the R_p_ value of the uncoated sample and the coated sample, respectively. The average R_p_ value (9793.747 Ω·cm^2^) of the coated sample is much larger than that (1434.000 Ω·cm^2^) of the uncoated sample, suggesting that the coated sample has better corrosion resistance.

Figure 13 presents the electrochemical impedance spectra of the uncoated and coated samples after being soaked in simulated concrete pore solutions with and without Cl^−^ for 1 h. The low-frequency impedance of the coated sample in both simulated concrete pore solutions is higher than that of the uncoated sample, indicating that the coated sample is more corrosion-resistant than the uncoated one. In addition, the low-frequency impedance of the sample in simulated concrete pore solution with Cl^−^ is smaller than that in simulated concrete pore solution without Cl^−^, suggesting that Cl^−^ can accelerate the corrosion of the magnesium–neodymium alloy. It can be seen in Figure 13c that the Nyquist plots of the samples all contain a high-frequency capacitive loop, while the Nyquist plots of the uncoated samples in a simulated concrete pore solution with Cl^−^ contain an extra low-frequency inductive loop. As for the size of the capacitive loops, all samples in the simulated concrete pore solution without Cl^−^ have bigger sizes that the ones in the simulated concrete pore solution with Cl^−^, and each coated sample has bigger sizes that the corresponding one. Figure 13d shows the equivalent circuit models for fitting the EIS data and the meaning of each electronic component is referred to the aforementioned introduction. Table 6 gives the results of the fitting and, based on the data, the polarization resistance is further calculated for evaluating corrosion resistance. Here, $R_{ct}$ and $R_{pore}+R_{ct}$ are suggested to calculate the R_p_ value of the uncoated sample and the coated sample in the simulated concrete pore solution without Cl^−^, respectively. The uncoated sample has an average R_p_ value of (1.431 ± 0.187) × 10^5^ Ω·cm^2^, which is similar to the coated sample’s average R_p_ value of (1.636 ± 0.270) × 10^5^ Ω·cm^2^. $\frac{R_{ct}R_{L}}{R_{ct}+R_{L}}$ and $R_{pore}+R_{ct}$ are used to calculate the R_p_ value of the uncoated sample and the coated sample in the simulated concrete pore solution with Cl^−^, respectively. As expected, the average R_p_ value (2498.923 Ω·cm^2^) of the coated sample is larger than that (1111.527 Ω·cm^2^) of the uncoated one.

**Figure 14:**
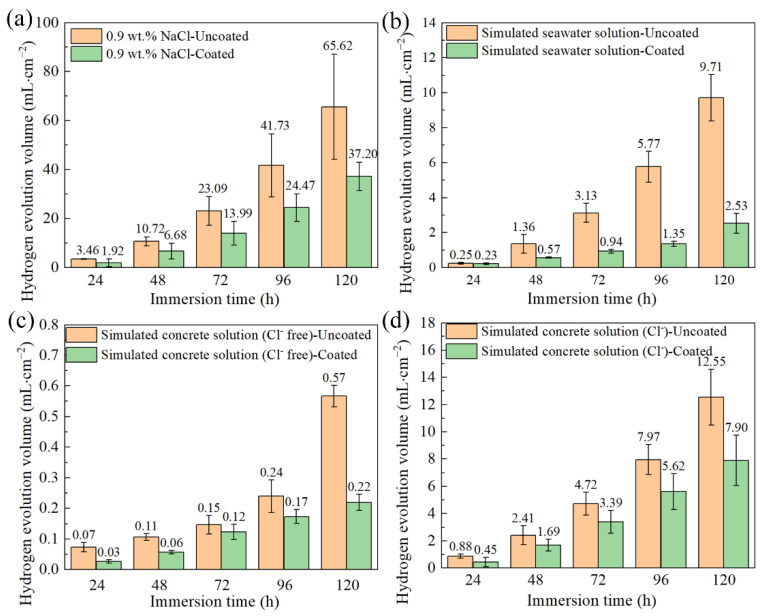
Hydrogen evolution volume of the investigated samples after immersion in different solutions for 120 h: (**a**) 0.9 wt.% NaCl solution at 37 °C, (**b**) simulated seawater solution, (**c**) simulated concrete pore solution without Cl^−^, and (**d**) simulated concrete pore solution with Cl^−^.

The hydrogen evolution of the coated and uncoated samples in three different solutions is shown in Figure 14. As shown in Figure 14a,b, the hydrogen evolution volumes of the coated samples in both the 0.9 wt.% NaCl solution and the simulated seawater solution are significantly smaller than those of the uncoated samples, indicating an enhanced corrosion resistance of the coated samples in those solutions. Figure 14c shows the hydrogen evolution of the investigated samples in the simulated concrete pore solution without Cl^−^. During the 120 h investigated, the hydrogen evolution volumes of both kinds of samples were very small, indicating that the sample has long-term corrosion resistance in the simulated concrete pore solution without Cl^−^. Figure 14d shows the hydrogen evolution of the investigated samples in the simulated concrete pore solution with Cl^−^. Comparing Figure 14c with Figure 14d, the hydrogen evolution volumes obtained in the simulated concrete pore solution containing Cl^−^ are much higher than those obtained in the simulated concrete pore solution without Cl^−^. This indicates that Cl^−^ can aggravate the corrosion of magnesium alloys in the simulated concrete pore solution. Fortunately, the coating has a certain protective effect against the attack from Cl^−^, which is validated by the results of the hydrogen evolution test and electrochemical evaluation.

**Figure 15:**
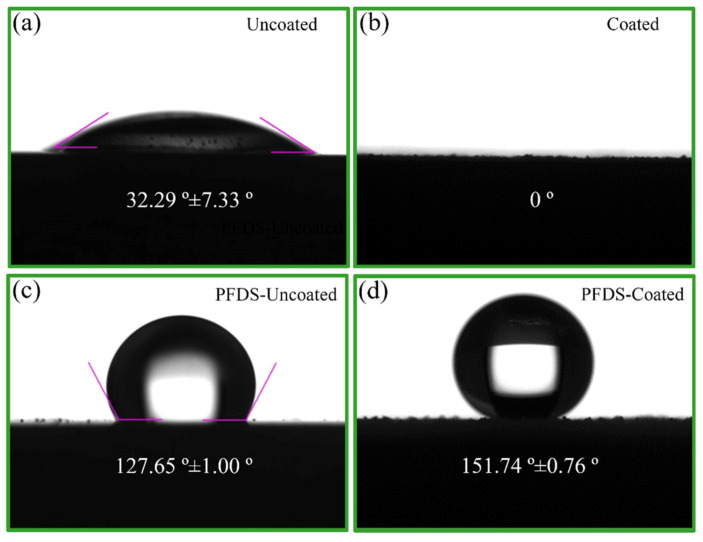
Contact angles of the investigated samples in this study: (**a**) uncoated, (**b**) coated, (**c**) PFDS-uncoated and (**d**) PFDS-coated.

**Figure 16:**
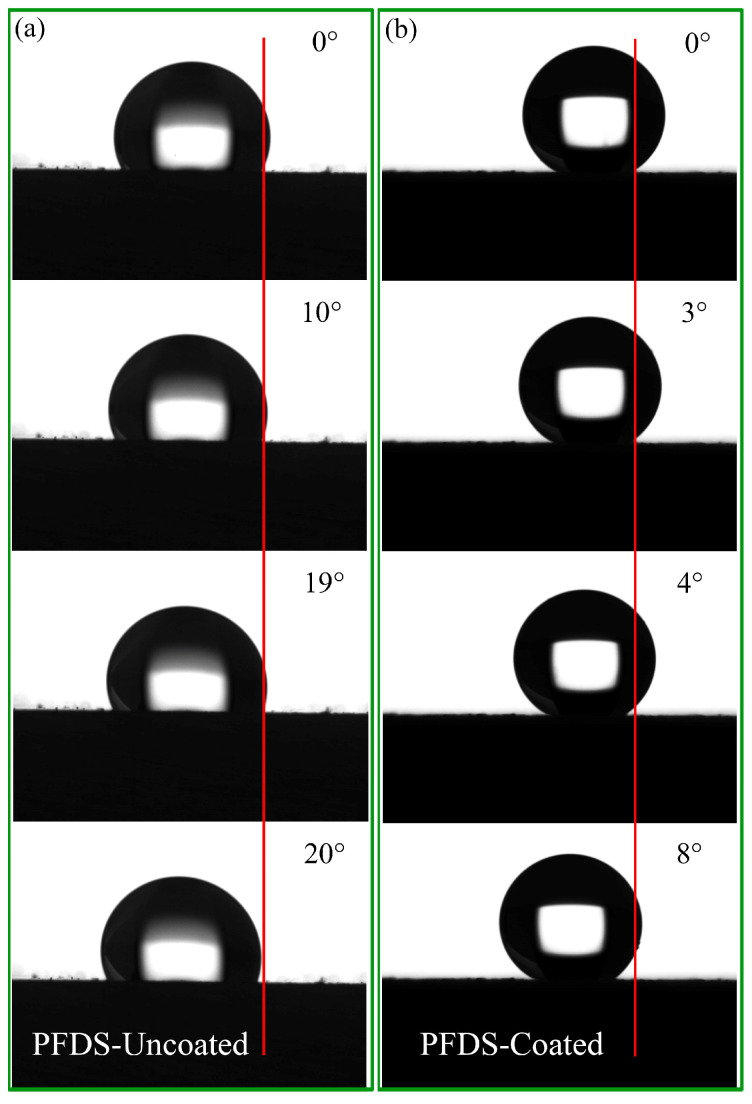
Sliding angles of the investigated samples in this study: (**a**) PFDS-uncoated, (**b**) PFDS-coated.

The static water contact angle and sliding angle are applied to evaluate the wettability of the samples. Figure 15 gives the static water contact angles of the uncoated and coated samples before and after surface fluorination. Before chemical surface modification, the static water contact angles of the uncoated and coated samples are 32.29 ± 7.33° and 0°, respectively, indicating the hydrophilic nature of their surfaces. After surface chemical modification, the static water contact angles of the uncoated and coated samples significantly increase, reaching 127.65 ± 1.00° and 151.74 ± 0.76°, respectively. Figure 16 shows the captured view of the movement of water droplets on the uncoated and coated samples after surface fluorination. As for the uncoated sample, the water droplet on it does not start moving until the tilt angle becomes 20°. But for the coated sample, the droplet starts rolling when the tilt angle is only about 4°. Combined with the results of the static water contact angle, it is concluded that, after surface modification, the coated sample achieved a superhydrophobic effect, which is believed to endow the surface with water-repellent and self-cleaning abilities [42].

## 4. Conclusions

In this study, a composite coating is successfully prepared on a magnesium–neodymium alloy by a simple chemical conversion process using a mixed solution of Ca(OH)_2_ and NaHCO_3_. The composite coating with two spontaneously formed layers is mainly composed of Mg(OH)_2_, CaCO_3_, and Mg_5_(OH)_2_(CO_3_)_4_∙4H_2_O. It is speculated that the top flower-like layer is composed of Mg_5_(OH)_2_(CO_3_)_4_∙4H_2_O, Mg(OH)_2_ and CaCO_3_, and the inner dense layer is possible to be Mg(OH)_2_. According to the results of electrochemical impedance spectroscopy, polarization test, and hydrogen evolution, it is determined that the coating can mitigate the corrosion of the Mg-Nd alloy effectively in three kinds of different solutions, including simulated physiological fluids, simulated sea water, and a simulated concrete pore solution. A simple chemical modification with fluorinated silane is further conducted to tailor the wettability of the coated sample, resulting in a superhydrophobic surface with an average water contact angle of 151.74° and a sliding angle of about 4°. In summary, a protection- and function-integrated coating can be achieved by the formation of a self-layered chemical conversion coating.

## Figures and Tables

**Table 1:** *E_corr_* and *I_corr_* calculated from the corresponding polarization curves obtained after immersion in 0.9 wt.% NaCl solution at 37 °C for 1 h.

	*E_corr_* (V/SCE)	*I_corr_* (A∙cm^−2^)
Uncoated	−1.541 ± 0.011	(1.330 ± 0.072) × 10^−4^
Coated	−1.414 ± 0.013	(7.548 ± 1.490) × 10^−6^

**Table 2:** *E_corr_* and *I_corr_* of samples calculated from the corresponding polarization curves after immersion in simulated seawater solution for 1 h.

	*E_corr_* (V/SCE)	*I_corr_* (A∙cm^−2^)
Uncoated	−1.659 ± 0.003	(5.310 ± 0.019) × 10^−4^
Coated	−1.541 ± 0.010	(8.650 ± 0.615) × 10^−6^

**Table 3:** *E_corr_* and *I_corr_* calculated from the corresponding polarization curves after immersion in simulated concrete pore solution for 1 h.

	*E_corr_* (V/SCE)	*I_corr_* (A∙cm^−2^)
Uncoated (Cl^−^ free)	−1.413 ± 0.008	(6.092 ± 0.801) × 10^−7^
Coated (Cl^−^ free)	−1.375 ± 0.001	(4.444 ± 0.152) × 10^−7^
Uncoated (Cl^−^)	−1.516 ± 0.031	(3.147 ± 0.816) × 10^−5^
Coated (Cl^−^)	−1.359 ± 0.026	(5.414 ± 0.664) × 10^−6^

**Table 4:** Fitted data of the samples in 0.9 wt.% NaCl solution for 1 h at 37 °C based on the corresponding equivalent circuit models.

	Uncoated	Coated
*R_s_* (Ω∙cm^2^)	33.127 ± 3.614	35.897 ± 0.808
*Y*_0,*f*_ (Ω^−1^∙cm^−2^∙s^n^)	-	(5.434 ± 1.239) × 10^−6^
*n_f_*	-	0.700 ± 0.007
*R_pore_* (Ω∙cm^2^)	-	57.270 ± 6.850
*Y*_0,*dl*_ (Ω^−1^∙cm^−2^∙s^n^)	(2.754 ± 0.491) × 10^−5^	(1.668 ± 0.193) × 10^−5^
*n_dl_*	0.922 ± 0.010	0.691 ± 0.009
*R_ct_* (Ω∙cm^2^)	232.800 ± 24.447	(4.436 ± 0.495) × 10^3^
*L* (H∙cm^2^)	550.533 ± 101.946	-
*R_L_* (Ω∙cm^2^)	151.633 ± 24.908	-

**Table 5:** Fitted data of the samples in simulated seawater solution for 1 h based on the corresponding equivalent circuit models.

	Uncoated	Coated
*R_s_* (Ω∙cm^2^)	19.493 ± 5.007	20.067 ± 8.183
*Y*_0,*f*_ (Ω^−1^∙cm^−2^∙s^n^)	-	(7.825 ± 6.382) × 10^−6^
*n_f_*	-	0.679 ± 0.071
*R_pore_* (Ω∙cm^2^)	-	64.747 ± 20.530
*Y*_0,*dl*_ (Ω^−1^∙cm^−2^∙s^n^)	(1.014 ± 0.215) × 10^−3^	(4.262 ± 2.370) × 10^−6^
*n_dl_*	0.721 ± 0.042	0.867 ± 0.124
*R_ct_* (Ω∙cm^2^)	574.900 ± 54.277	(3.326 ± 0.411) × 10^3^
*Y*_0,*diff*_ (Ω^−1^∙cm^−2^∙s^n^)	(1.425 ± 0.063) × 10^−5^	(4.591 ± 0.842) × 10^−5^
*n_diff_*	0.923 ± 0.003	0.719 ± 0.082
*R_diff_* (Ω∙cm^2^)	859.100 ± 76.464	(6.403 ± 0.423) × 10^3^

**Table 6:** Fitted data of the samples in simulated concrete pore solution for 1 h based on the corresponding equivalent circuit models.

	Uncoated (Cl^−^ Free)	Coated (Cl^−^ Free)	Uncoated (Cl^−^)	Coated (Cl^−^)
*R_s_* (Ω∙cm^2^)	113.833 ± 8.014	157.733 ± 9.880	17.730 ± 6.760	12.330 ± 0.771
*Y*_0,*f*_ (Ω^−1^∙cm^−2^∙s^n^)	-	(7.792 ± 1.517) × 10^−6^	-	(6.801 ± 0.897) × 10^−6^
*n_f_*	-	0.770 ± 0.044	-	0.821 ± 0.018
*R_pore_* (Ω∙cm^2^)	-	(1.960 ± 0.549) × 10^4^	-	62.923 ± 17.766
*Y*_0,*dl*_ (Ω^−1^∙cm^−2^∙s^n^)	(9.776 ± 0.440) × 10^−6^	(7.236 ± 1.286) × 10^−6^	(2.018 ± 0.208) × 10^−5^	(4.893 ± 0.095) × 10^−6^
*n_dl_*	0.913 ± 0.001	0.709 ± 0.026	0.882 ± 0.007	0.815 ± 0.006
*R_ct_* (Ω∙cm^2^)	(1.431 ± 0.187) × 10^5^	(1.440 ± 0.215) × 10^5^	(1.540 ± 0.304) × 10^3^	(2.436 ± 0.242) × 10^3^
*L* (H∙cm^2^)	-	-	177.700 ± 58.613	-
*R_L_* (Ω∙cm^2^)	-	-	(3.995 ± 0.131) × 10^3^	-

## Data Availability

The original contributions presented in the study are included in the article, further inquiries can be directed to the corresponding author.
